# The H_2_FPEF and HFA-PEFF algorithms for predicting exercise intolerance and abnormal hemodynamics in heart failure with preserved ejection fraction

**DOI:** 10.1038/s41598-021-03974-6

**Published:** 2022-01-07

**Authors:** Shiro Amanai, Tomonari Harada, Kazuki Kagami, Kuniko Yoshida, Toshimitsu Kato, Naoki Wada, Masaru Obokata

**Affiliations:** 1 Department of Cardiovascular Medicine, Gunma University Graduate School of Medicine, 3-39-22 Showa-machi, Maebashi, Gunma 371-8511 Japan; 2 Division of Cardiovascular Medicine, National Defense Medical College, Tokorozawa, Saitama Japan; 3 Department of Rehabilitation Medicine, Gunma University Graduate School of Medicine, Maebashi, Gunma Japan

**Keywords:** Cardiology, Heart failure

## Abstract

Exercise intolerance is a primary manifestation in patients with heart failure with preserved ejection fraction (HFpEF) and is associated with abnormal hemodynamics and a poor quality of life. Two multiparametric scoring systems have been proposed to diagnose HFpEF. This study sought to determine the performance of the H_2_FPEF and HFA-PEFF scores for predicting exercise capacity and echocardiographic findings of intracardiac pressures during exercise in subjects with dyspnea on exertion referred for bicycle stress echocardiography. In a subset, simultaneous expired gas analysis was performed to measure the peak oxygen consumption (VO_2_). Patients with HFpEF (n = 83) and controls without HF (n = 104) were enrolled. The H_2_FPEF score was obtainable for all patients while the HFA-PEFF score could not be calculated for 23 patients (feasibility 88%). Both H_2_FPEF and HFA-PEFF scores correlated with a higher E/e′ ratio (r = 0.49 and r = 0.46), lower systolic tricuspid annular velocity (r =  − 0.44 and =  − 0.24), and lower cardiac output (r =  − 0.28 and r =  − 0.24) during peak exercise. Peak VO_2_ and exercise duration decreased with an increase in H_2_FPEF scores (r =  − 0.40 and r =  − 0.32). The H_2_FPEF score predicted a reduced aerobic capacity (AUC 0.71, *p* = 0.0005), but the HFA-PEFF score did not (*p* = 0.07). These data provide insights into the role of the H_2_FPEF and HFA-PEFF scores for predicting exercise intolerance and abnormal hemodynamics in patients presenting with exertional dyspnea.

## Introduction

Heart failure (HF) with preserved ejection fraction (HFpEF) accounts for more than half of all the patients diagnosed with HF. There is a worldwide increase in its prevalence with limited proven treatment, making it an important healthcare problem of modern cardiology^1^. Exercise intolerance is a primary manifestation in patients with HFpEF and is associated with symptoms of dyspnea, abnormal central hemodynamics, and a poor quality of life^2–4^. The identification of reduced aerobic capacity may have potentially important therapeutic implications in people with HFpEF.

Recent studies have proposed two multiparametric scoring systems to help diagnose HFpEF in patients with dyspnea. First, the H_2_FPEF score that is based on four clinical variables (the body mass index [BMI], use of two or more antihypertensive medicines, atrial fibrillation [AF], and age) and two echocardiographic variables (the ratio of the early diastolic mitral inflow velocity [E] to early diastolic mitral annular tissue velocity [e′] [E/e′ ratio] and pulmonary artery [PA] systolic pressure [PASP])^5^. Second, the HFA-PEFF score that employs a complex approach by scoring the natriuretic peptide levels and the echocardiographic findings of cardiac function and structure^6^. In addition to the diagnostic value, recent studies have demonstrated the association between the two algorithms and the clinical outcomes in patients diagnosed with HFpEF^7–10^. However, few data are available regarding whether these diagnostic schemes could predict aerobic capacity in patients with HFpEF.

Accordingly, we examined the performance of the two algorithms for predicting exercise capacity and the echocardiographic estimates of intracardiac pressures during ergometry exercise echocardiography.

## Results

### Subject characteristics

Of 187 participants, 83 patients were found to have HFpEF, and 104 had non-cardiac dyspnea (controls). Of the 83 patients with HFpEF, 26 patients were diagnosed through invasive catheterization, 24 with the American Society of Echocardiography/European Association of Cardiovascular Imaging (ASE/EACVI) criteria for diastolic dysfunction, and the remaining 33 due to an elevated E/e′ ratio during exercise. Comparisons of the clinical profiles, the HFpEF scores, and the exercise capacity across the different definitions of HFpEF are presented in Supplemental Table 1. The clinical characteristics of the 83 patients with HFpEF and 104 controls are shown in Table 1. As compared to the control groups, patients with HFpEF were older and had a higher prevalence of systemic hypertension, diabetes, coronary artery disease, AF, and elevated natriuretic peptide levels. Sex, BMI, and vital signs were similar between the two groups. Patients with HFpEF were treated with angiotensin-converting enzyme inhibitors or angiotensin-receptor blockers, beta-blockers, and loop diuretics more frequently than the control group.

**Table 1:** Baseline characteristics.

	Controls (n = 104)	HFpEF (n = 83)	*P* value
Age (years)	63 ± 13	74 ± 8	< 0.0001
Female, n (%)	68 (65%)	50 (60%)	0.47
Body mass index (kg/m^2^)	23.1 ± 5.0	24.4 ± 4.2	0.06
**Comorbidities**			
Coronary disease, n (%)	5 (5)	12 (15)	0.02
Diabetes mellitus, n (%)	12 (12)	20 (24)	0.03
Hypertension, n (%)	69 (66)	67 (81)	0.03
Atrial fibrillation, n (%)	10 (10)	31 (37)	< 0.0001
**Medications**			
ACEI or ARB, n (%)	30 (29)	38 (46)	0.02
Beta-blocker, n (%)	9 (9)	31 (37)	< 0.0001
Loop diuretics, n (%)	15 (14)	28 (34)	0.002
**Laboratories**			
BNP (pg/mL), n = 128	34 (17, 60)	98 (39,158)	< 0.0001
NT-proBNP (pg/mL), n = 83	99 (66, 153)	558 (152, 1378)	< 0.0001
**Vital signs**			
Heart rate (bpm)	76 ± 14	73 ± 15	0.12
Systolic BP (mmHg)	130 ± 23	130 ± 20	0.81
Saturation (%)	97 ± 2	97 ± 1	0.52
**LV and LA structures**			
LV diastolic dimension (mm)	43 ± 5	44 ± 6	0.08
LV mass index (g/m^2^)	79 ± 19	93 ± 23	< 0.0001
Relative wall thickness	0.43 ± 0.08	0.46 ± 0.11	0.02
LA volume index (mL/m^2^)	24 (19, 31)	39 (30, 50)	< 0.0001
**LV and RV function**			
LV ejection fraction (%)	64 ± 7	63 ± 7	0.21
LV longitudinal strain (%), n = 172	17.7 ± 3.1	15.4 ± 3.7	< 0.0001
E-wave (cm/sec)	66 ± 18	85 ± 27	< 0.0001
Mitral septal e′ (cm/sec)	7.0 ± 2.5	5.4 ± 2.3	< 0.0001
Mitral septal s′ (cm/sec)	7.6 ± 1.5	6.5 ± 1.7	< 0.0001
Mitral lateral e′ (cm/sec)	9.4 ± 3.2	7.2 ± 2.9	< 0.0001
Mitral lateral s′ (cm/sec)	9.2 ± 2.5	7.6 ± 2.1	< 0.0001
E/e′ ratio (average)	8.6 ± 2.6	14.2 ± 7.9	< 0.0001
Cardiac output (L/min)	4.2 ± 1.3	4.0 ± 1.0	0.30
A-VO_2_ diff (mL/dL)	6.1 ± 2.3	6.3 ± 2.2	0.65
TV s′ (cm/sec)	12.6 ± 3.0	11.6 ± 3.3	0.04
PASP (mmHg)	20 ± 6	24 ± 9	< 0.0001
RAP (mmHg)	3 ± 1	5 ± 3	0.0004
**HFpEF Scores**			
H_2_FPEF score, n = 187	1.8 ± 1.3	3.6 ± 1.7	< 0.0001
HFA-PEFF score, n = 164	3.1 ± 1.3	4.8 ± 1.3	< 0.0001
H_2_FPEF score, low/intermediate/high (%)	46%/54%/0%	4%/79%/17%	< 0.0001
HFA-PEFF score, low/intermediate/high (%)	11%/76%/13%	0%/34%/66%	< 0.0001

Data are mean ± SD, median (interquartile range), or n (%).

ACEI, angiotensin-converting enzyme inhibitors; ARB, angiotensin-receptor blockers; A-VO_2_ diff, arterial-venous oxygen content difference; BNP, B-type natriuretic peptide; BP, blood pressure; E/e′ ratio, the ratio of early diastolic mitral inflow to mitral annular tissue velocities; HFpEF, heart failure with preserved ejection fraction; LA, left atrial; LV, left ventricular; NT-proBNP, N-terminal pro B-type natriuretic peptide; PASP, pulmonary artery systolic pressure; RAP, right atrial pressure; RV, right ventricular; and TV, tricuspid valvular.

As per resting echocardiography, patients with HFpEF had a larger left ventricular (LV) mass index, relative wall thickness, and left atrial (LA) volume index and poorer LV shortening evidenced by lower longitudinal strain and the systolic mitral annular tissue velocity at the septal annulus (mitral s′) compared to control subjects (Table 1). The LV diastolic function was poorer in patients with HFpEF, with lower mitral e′ velocity and a higher E/e′ ratio as compared to the controls. Patients with HFpEF displayed a higher estimated PASP and right atrial pressure (RAP) and lower velocity at the lateral tricuspid annulus (TV s′) than controls. EF, cardiac output, and arterial-venous oxygen content difference (A-VO_2_ diff) did not differ between the groups.

### The H_2_FPEF and HFA-PEFF scores

**Figure 1:**
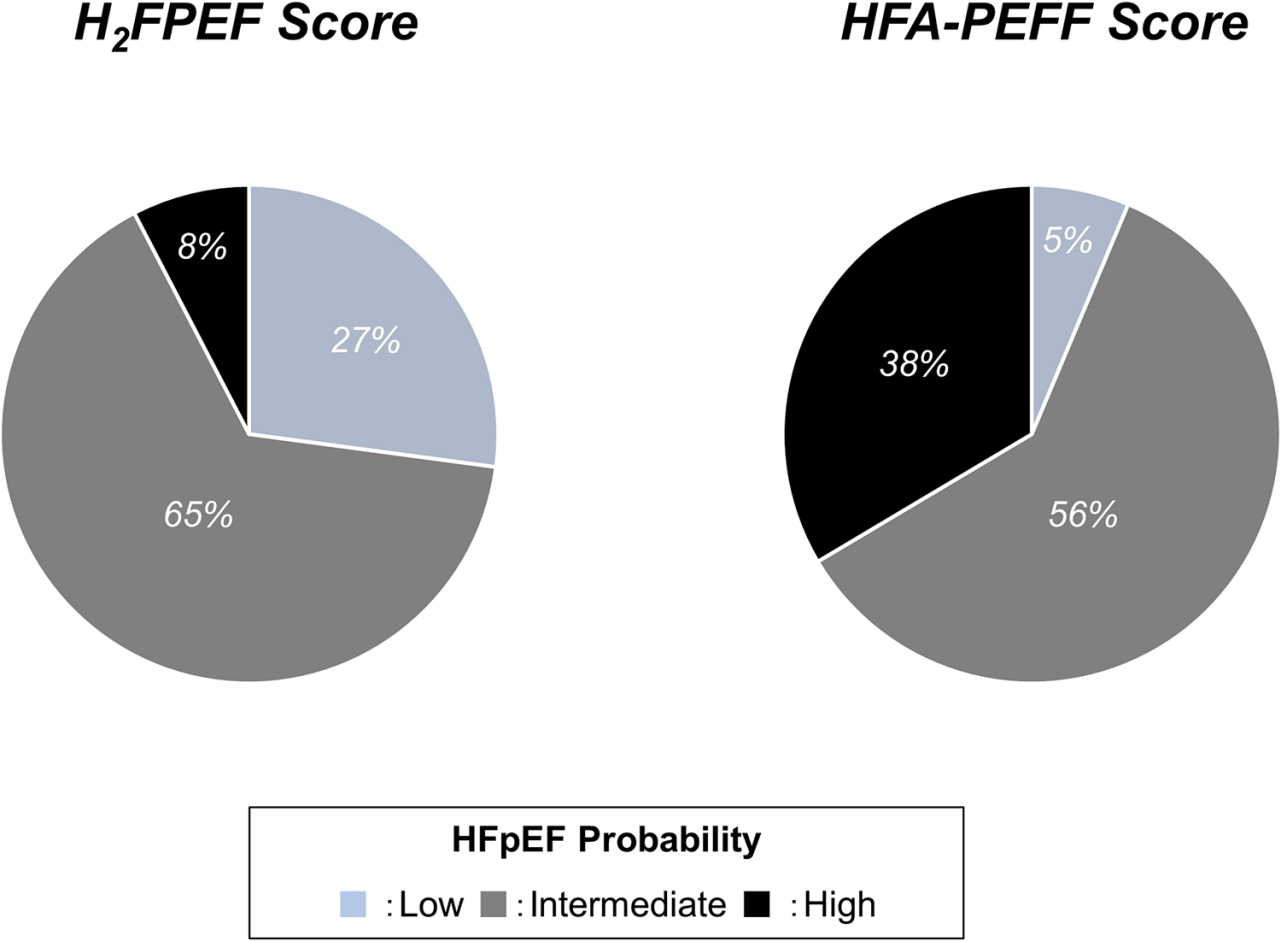
Distribution of H_2_FPEF and HFA-PEFF scores among all participants. The H_2_FPEF score was more likely to classify subjects into a low or intermediate probability while the HFA-PEFF score categorized them as a high probability.

Since data on E/e′ ratio and PASP were available in all participants, the H_2_FPEF score was calculated for all the patients enrolled in this study; however, the HFA-PEFF score could not be calculated for 23 patients (feasibility, 88%) because of missing data for natriuretic peptide levels. Among the entire cohort, subjects were likely to be classified as low or intermediate probabilities based on the H_2_FPEF score and as high probability based on the HFA-PEFF score (Fig. 1). As expected, both H_2_FPEF and HFA-PEFF scores were higher for patients with HFpEF than controls (Table 1). Both high H_2_FPEF (6–9 points) and HFA-PEFF (5–6 points) scores showed very high positive predictive values (100% and 83%) to diagnose HFpEF while low scores (H2FPEF: 0–1 points and HFA-PEFF: 0–1 points) displayed very high negative predictive values (94% and 100%) (Supplemental Table 2).

### Exercise capacity and echocardiographic findings during peak exercise

**Figure 2:**
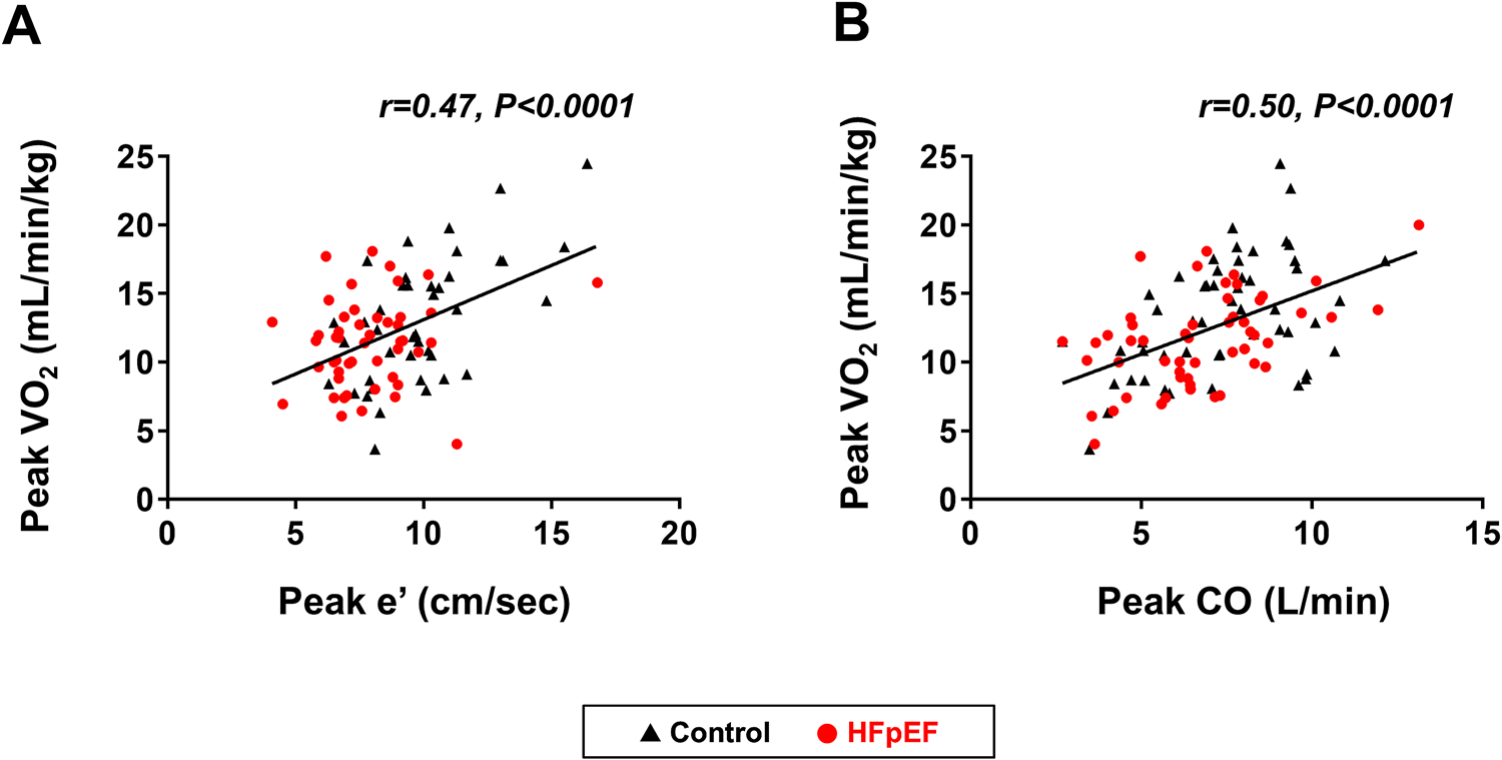
Correlations between echocardiographic measures and exercise capacity. Decreases in mitral annular e′ velocity and cardiac output (CO) during exercise were moderately correlated with lower peak oxygen consumption (VO_2_). HFpEF, heart failure with preserved ejection fraction (HFpEF).

An expired gas analysis was performed simultaneously in 53 patients with HFpEF and 54 control subjects. As compared to the controls, peak exercise workload was lower, exercise duration was shorter, and peak VO_2_ was lower in HFpEF patients (Table 2). During the period of peak exercise, heart rate was lower in patients with HFpEF as compared to the controls while the systolic blood pressure, oxygen saturation, and A-VO_2_ diff were similar between the groups. Differences in the mitral e′ and s′ tissue velocities and the E/e′ ratio between the groups increased further during the peak exercise. Compared to the control subjects, HFpEF patients had a lower cardiac output (CO) and TV s′ and a higher PASP during the peak exercise. The severity of the e′ and s′ velocities, E/e′ ratio, CO, A-VO_2_ diff, and TV s′ were consistently associated with poor exercise capacity (Fig. 2 and Table 3).

**Table 2:** Exercise capacity and echocardiographic measures during peak exercise.

	Controls (n = 104)	HFpEF (n = 83)	*P* value
**Exercise capacity**			
Peak watts (W)	63 ± 24	50 ± 23	0.0003
Exercise time (min)	10.2 ± 3.3	8.6 ± 3.2	0.001
Peak VO_2_ (mL/min/kg), n = 107	13.3 ± 4.3	11.6 ± 3.3	0.03
**Vital signs**			
Heart rate (bpm)	118 ± 21	111 ± 24	0.04
Systolic BP (mmHg)	167 ± 32	161 ± 33	0.20
Saturation (%)	94 ± 4	95 ± 4	0.68
**Echocardiographic measures**			
LV ejection fraction (%)	72 ± 8	69 ± 9	0.03
E-wave (cm/sec)	106 ± 24	129 ± 30	< 0.0001
Mitral e′ (cm/sec)	10.0 ± 2.5	7.4 ± 2.0	< 0.0001
Mitral s′ (cm/sec)	9.3 ± 2.3	7.3 ± 2.2	< 0.0001
E/e′ ratio (septal)	11.0 ± 2.6	18.4 ± 6.3	< 0.0001
Cardiac output (L/min)	7.8 ± 2.2	6.6 ± 2.1	0.0003
A-VO_2_ diff (mL/dL)	10.7 ± 4.4	11.2 ± 4.3	0.56
TV s′ (cm/sec)	15.2 ± 3.0	12.8 ± 3.6	< 0.0001
PASP (mmHg)	37 ± 11	45 ± 12	< 0.0001

Data are mean ± SD or median (interquartile range). VO_2_, oxygen consumption, and other abbreviations as in Table 1.

**Table 3:** Correlations between echocardiographic measures of hemodynamics and exercise capacity.

	Exercise duration		Peak VO_2_	
	r coefficient	*P* value	r coefficient	*P* value
Mitral e′ (cm/sec)	0.47	< 0.0001	0.47	< 0.0001
Mitral s′ (cm/sec)	0.44	< 0.0001	0.29	0.0009
E/e′ ratio	− 0.32	< 0.0001	− 0.22	0.01
Cardiac output (L/min)	0.48	< 0.0001	0.50	< 0.0001
A-VO_2_ diff (mL/dL)	0.42	< 0.0001	0.54	< 0.0001
TV s′ (cm/sec)	0.41	< 0.0001	0.35	< 0.0001
PASP (mmHg)	− 0.01	0.90	0.15	0.15

Abbreviations as in Tables 1 and 2.

### Correlations of the HFpEF scores with the echocardiographic findings during peak exercise and the exercise capacity

The H_2_FPEF score correlated with the echocardiographic findings during peak ergometry exercise (Table 4). Particularly, a higher H_2_FPEF score was associated with a lower LV systolic function (s′ velocity, r =  − 0.46, *p* < 0.0001), higher E/e′ ratio (r = 0.49, *p* < 0.0001), and lower RV systolic function (TV s′, r =  − 0.44, *p* < 0.0001) during peak exercise. These correlations remained significant after adjusting for the age, sex, BMI, and presence of hypertension (all *p* < 0.05). There were similar correlations between the HFA-PEFF score and the echocardiographic findings (Table 4). After adjusting for the age, sex, BMI, and presence of hypertension, the HFA-PEFF score remained significantly associated with mitral e′ and s′ velocities and E/e′ ratio (*p* < 0.01); however, this was not the case for CO, TV s′, and PASP (*p* > 0.05). The H_2_FPEF and HFA-PEFF scores did not correlate with A-VO_2_ diff during exercise.

**Table 4:** Correlations of the two HFpEF Scores with exercise capacity and echocardiographic measures during peak exercise.

	H_2_FPEF score		HFA-PEFF score	
	r coefficient	*P* value	r coefficient	*P* value
**Echocardiographic measures during peak exercise**				
Mitral e′ (cm/sec)	− 0.35	< 0.0001	− 0.47	< 0.0001
Mitral s′ (cm/sec)	− 0.46	< 0.0001	− 0.47	< 0.0001
E/e′ ratio	0.49	< 0.0001	0.46	< 0.0001
Cardiac output (L/min)	− 0.28	< 0.0001	− 0.24	0.002
A-VO_2_ diff (mL/dL)	0.09	0.38	− 0.01	0.93
TV s′ (cm/sec)	− 0.44	< 0.0001	− 0.24	0.002
PASP (mmHg)	0.18	0.01	0.20	0.01

Abbreviations as in Tables 1 and 2.

**Figure 3:**
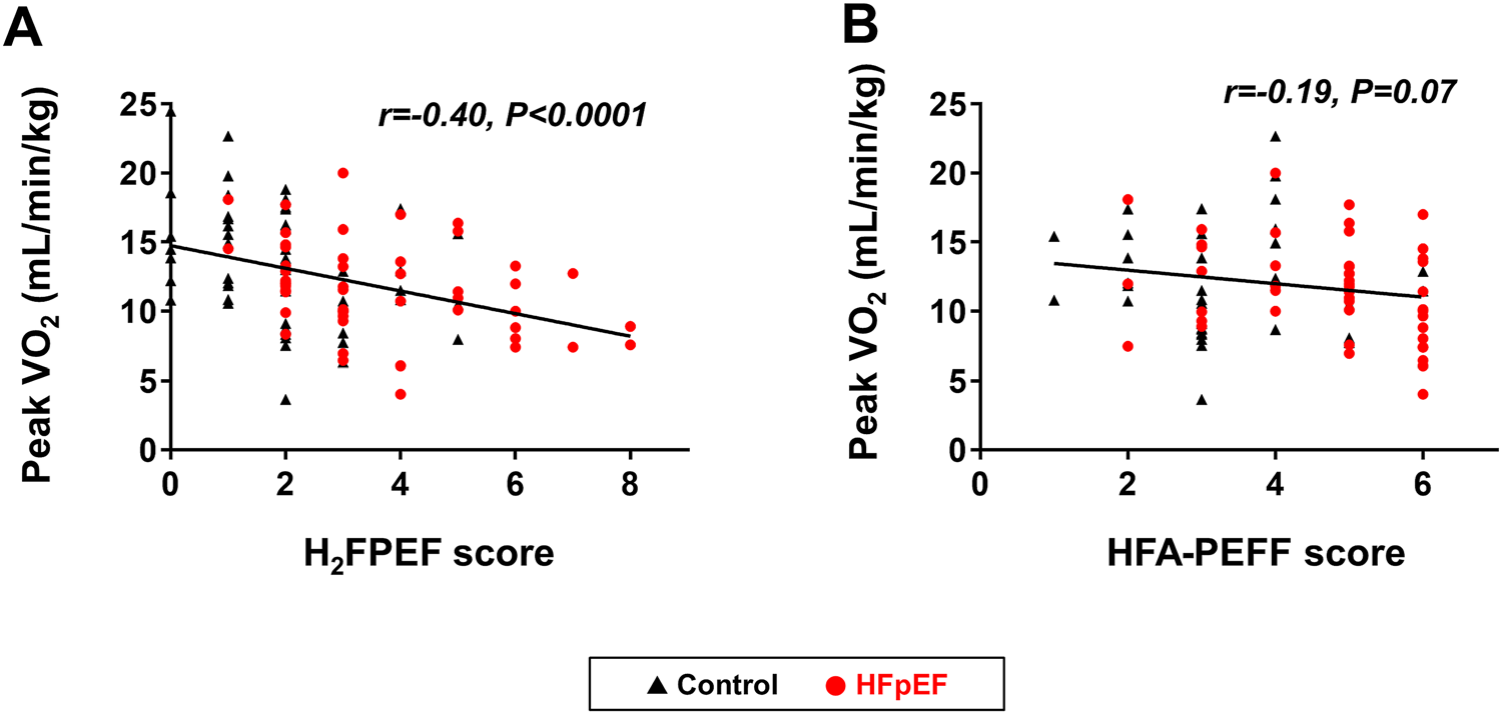
Correlations between HFpEF diagnostic algorithms and exercise capacity. The H_2_FPEF score was correlated with peak VO_2_, but the HFA-PEFF score was not. Abbreviations as in Fig. 2.

**Figure 4:**
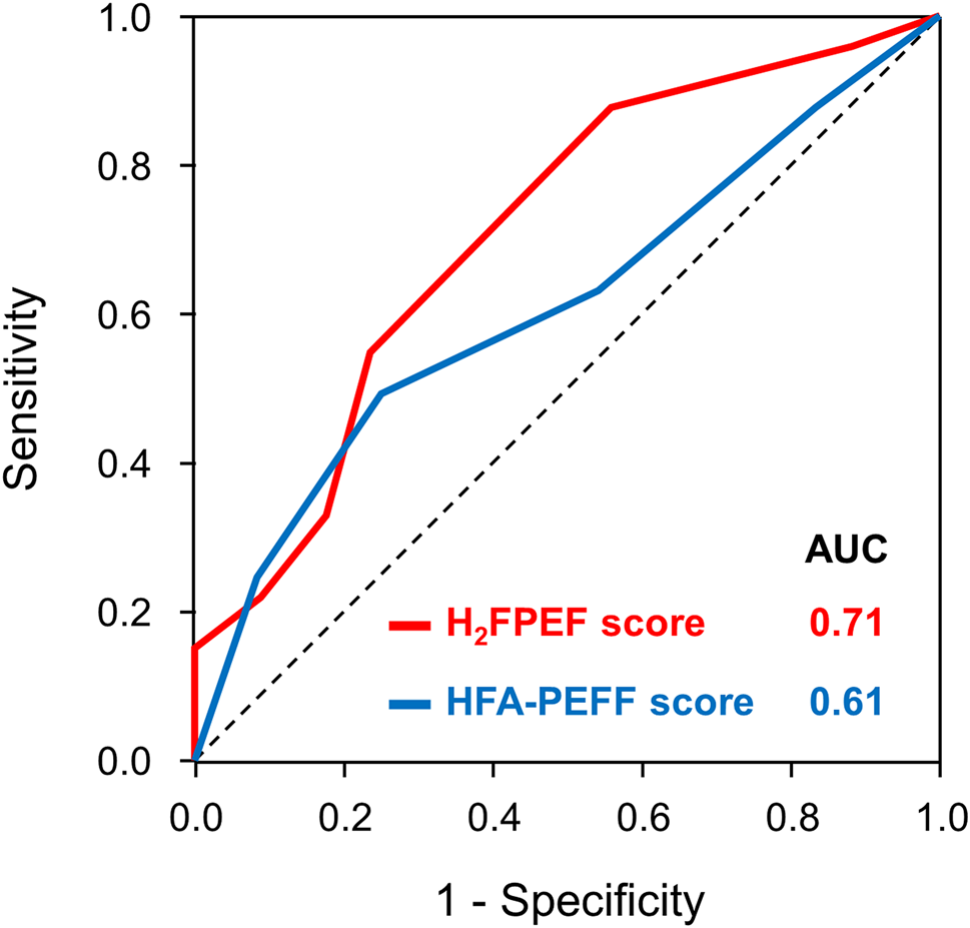
Receiver-operating characteristic curves of the H_2_FPEF and HFA-PEFF scores to predict impaired exercise capacity. AUC, area under the curve.

**Figure 5:**
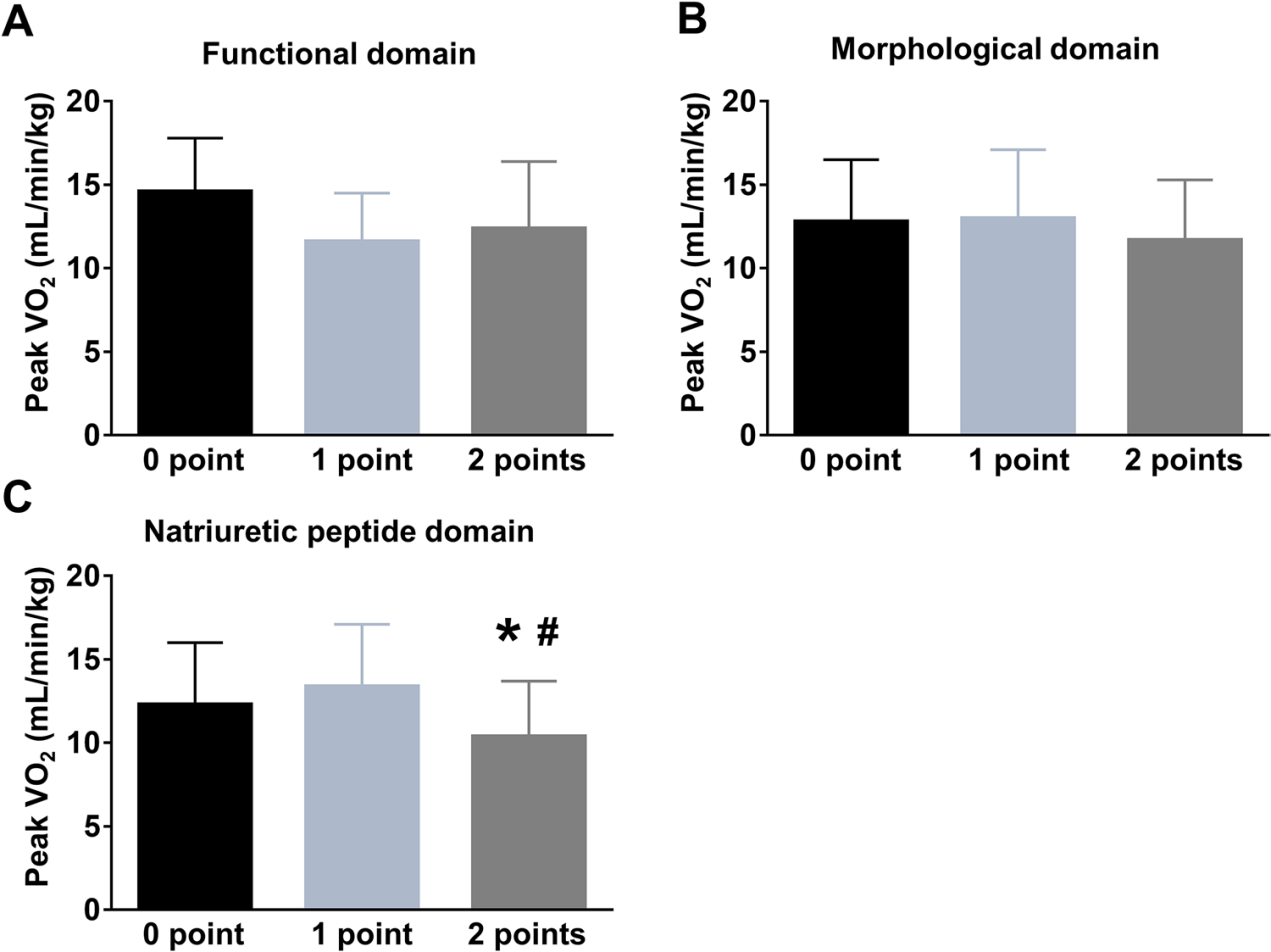
Peak oxygen consumption according to the HFA-PEFF domain scores. (**A**) Peak VO_2_ did not differ among the HFA-PEFF functional domain scores. (**B**) Peak VO_2_ was similar among the HFA-PEFF morphological domain scores. (**C**) In contrast, patients with a natriuretic peptide domain of 2 points displayed lower peak VO_2_ compared to the other groups. *p < 0.05 vs. 0 point, #*p* < 0.05 vs. 1 point. Abbreviations as in Fig. 2.

Peak VO_2_ and exercise duration decreased with an increase in the H_2_FPEF score (Fig. 3, r =  − 0.40 and − 0.32, *p* < 0.0001). As expected, both peak VO_2_ and exercise duration were related to age (r =  − 0.27, *p* = 0.005 and r =  − 0.41, *p* < 0.0001), and the H_2_FPEF score remained significantly associated with peak VO_2_ and the exercise time even after adjusting for the age, sex, BMI, and presence of hypertension (*p* = 0.03 and *p* = 0.0002). The H_2_FPEF score demonstrated a good discriminatory ability for identifying reduced aerobic capacity (peak VO_2_ < 14 mL/min/kg) (area under the curve [AUC] 0.71, 95% confidence interval [95%CI] 0.59–0.80, *p* = 0.0005: Fig. 4). An H_2_FPEF score of ≥ 6 displayed a high specificity (100%) and low sensitivity (15%) for the identification of reduced peak VO_2_, whereas an H_2_FPEF score of ≤ 1 had high sensitivity (96%) but very poor specificity (12%). Among components of the H_2_FPEF score, the age, BMI, E/e′ ratio, and treatment with 2 or more antihypertensives were related to the peak VO_2_ but they had modest relationships (Table 5). The HFA-PEFF score correlated with exercise duration (r =  − 0.26, *p* = 0.0008), but not with the peak VO_2_ (r =  − 0.19, *p* = 0.07). The natriuretic peptide level domain was the only component of the HFA-PEFF score associated with peak VO_2_ (Fig. 5). The HFA-PEFF score did not predict reduced aerobic capacity (AUC 0.61, 95%CI 0.49–0.73, *p* = 0.10: Fig. 4).

**Table 5:** Correlations of the components of H_2_FPEF and HFA-PEFF scores with exercise capacity.

	Peak VO_2_		Exercise time	
	r	P value	r	P value
**Components of the H**_**2**_**FPEF score**				
Age (years)	− 0.27	0.005	− 0.41	< 0.0001
Body mass index (kg/m^2^)	− 0.25	0.009	0.14	0.06
E/e′ ratio	− 0.23	0.02	− 0.30	< 0.0001
PASP (mmHg)	− 0.12	0.21	− 0.19	0.01
AF or SR	–	0.10*	–	0.06*
Hypertensive	–	0.01*	–	0.03*
**Components of the HFA-PEFF score**				
e′ (cm/sec)	0.16	0.10	0.32	< 0.0001
Longitudinal strain (%)	0.21	0.03	0.15	0.05
LA volume index (mL/m^2^)	− 0.16	0.11	− 0.18	0.01
LV mass index (g/m^2^)	− 0.01	0.91	− 0.13	0.07
Ln BNP (n = 65)	− 0.26	0.04	− 0.30	0.0006
Ln NT-proBNP (n = 30)	− 0.25	0.18	− 0.36	0.001

Abbreviations as in Tables 1 and 2.

*Determined by paired t-tests.

A sensitivity analysis performed for patients with HFpEF and the control subjects separately is presented in Supplemental Table 3. The H_2_FPEF score correlated with mitral s′, TV s′, peak VO_2_, and exercise duration in both HFpEF patients and controls; however, some of the correlations failed to remain significant possibly due to the small sample size. The HFA-PEFF score correlated with mitral e′ and s′ velocities, the E/e′ ratio, and exercise duration in the control subjects, and it correlated with mitral s′ and peak VO_2_ in patients with HFpEF. There were significant differences in correlations in the H_2_FPEF score with E/e′ ratio and TV s′ between HFpEF patients and controls (*p* = 0.02 and 0.04 by Meng’s z-test).

## Discussion

In this study, we examined the performance of the H_2_FPEF and HFA-PEFF scores for predicting exercise capacity and echocardiographic findings during exercise stress echocardiography. We observed that the HFA-PEFF score displayed relatively low feasibility because of the requirement of natriuretic peptide levels while the H_2_FPEF score displayed high feasibility. We further demonstrated that both H_2_FPEF and HFA-PEFF algorithms predicted a lower LV systolic and diastolic function, higher estimated LV filling pressure, lower CO, and reduced RV systolic function during peak ergometry exercise. Despite the requirement of a few input variables, the H_2_FPEF score provided a good discriminatory ability for identifying poor exercise capacity among patients with dyspnea while the HFA-PEFF score did not. These data provide new insights into the role of the H_2_FPEF and HFA-PEFF scores for predicting exercise intolerance and hemodynamic instability in patients presenting with exertional dyspnea.

Exercise intolerance is a hallmark in patients with HFpEF and studies have uniformly reported that peak VO_2_ is depressed in HFpEF^2,3,11–14^. Reduced exercise capacity is associated with symptoms of dyspnea and a poor quality of life, making it an important therapeutic target in this syndrome^3,15^. While multiple mechanisms can contribute to exercise intolerance in HFpEF, including abnormalities in the heart, lungs, and the periphery, a pathological increase in cardiac filling pressure developed during exercise stress may play an important role in limiting exercise performance in patients with HFpEF^2,3,16–18^. The current study demonstrated that the exercise capacity was reduced in patients with HFpEF compared to control subjects as shown by a lower peak exercise workload, shorter exercise duration, and decrease in the peak VO_2_. Compared to the controls, patients with HFpEF displayed lower mitral e′ and s′ tissue velocities, a higher E/e′ ratio, reduced CO, and lower TV s′ during the peak exercise and the severity of these abnormalities were consistently associated with a decrease in exercise capacity. These data confirm that abnormal exercise hemodynamics and a reduced CO reserve contribute to exercise intolerance in patients with HFpEF.

Two multiparametric scoring systems have been proposed to help diagnose HFpEF among patients with dyspnea. The H_2_FPEF score is developed among patients with unexplained dyspnea using the currently-recommended gold standard test (i.e., invasive exercise hemodynamic testing), and combines clinical characteristics and echocardiographic measures^5^. The HFA-PEFF score is a consensus-based approach that employs complex scoring systems based on echocardiographic indices and natriuretic peptide levels^6^. In the current study, the H_2_FPEF score was calculated for all patients while the HFA-PEFF score could not be calculated for 12% of the patients due to the lack of data for the natriuretic peptide levels. Although evaluation of the natriuretic peptide levels is a vital part of the diagnostic approach for HFpEF, they may not be measured in all patients with dyspnea^6^. On the contrary, echocardiography is performed in essentially all patients with a clinical suspicion for HFpEF^19^. It is easier for clinicians to calculate the H_2_FPEF score, with only a few variables than the HFA-PEFF score. Our data suggest that the H_2_FPEF score may have great significance in clinical practice. The current study also demonstrated that both high H_2_FPEF and HFA-PEFF scores showed very high positive predictive values (100% and 83%) to diagnose HFpEF while low scores displayed very high negative predictive values (94% and 100%). These data are consistent with the concept of the HFpEF scoring schemes^5,6^, and exercise stress testing is not required in the patients with very high or low scores. In patients with intermediate probability, exercise testing will be required. Further studies are warranted to directly compare the diagnostic value between the two scores using the gold standard of invasive exercise hemodynamic testing.

In addition to the diagnostic value, recent studies have shown the association between the two algorithms and the clinical outcomes in patients with HFpEF^7–10^. However, few data are available regarding whether these diagnostic schemes could predict aerobic capacity in HFpEF. In this study, we found that the H_2_FPEF score was associated with a reduced exercise capacity as assessed by both peak VO_2_ and exercise duration, which is consistent with the findings of a previous study^20^. In contrast, while the HFA-PEFF score modestly correlated with the exercise duration (r =  − 0.26), it was unrelated to the peak VO_2_. We further demonstrated that the H_2_FPEF score identified a reduction in the peak VO_2_; however, the HFA-PEFF algorithm did not. The plausible reason for this may be related to the inclusion of both clinical and echocardiographic variables in the H_2_FPEF score, but not in the HFA-PEFF score. It is clear that HFpEF is associated with comorbidities, including obesity, hypertension, diabetes, and AF^12–15^. Previous studies have demonstrated that a higher BMI and AF are related to decreased exercise capacity in patients with HFpEF^12,14,21,22^. The present study showed that individual components of the H_2_FPEF score were associated with peak VO_2_. In contrast, only LV longitudinal strain and B-type natriuretic peptides correlated with peak oxygen consumption among the components of the HFA-PEFF score. It is also worth pointing out that the H_2_FPEF score as a whole is more predictive of the peak VO_2_ than the individual components. These data support the usefulness of the H_2_FPEF score for not only diagnosing HFpEF but also for predicting exercise capacity and worse hemodynamics during exercise.

Reduced exercise intolerance is a cardinal manifestation in patients with HFpEF. Given its association with symptoms of dyspnea, abnormal central hemodynamics, and a poor quality of life^2–4^, impaired exercise capacity may reflect the severity of the HFpEF syndrome. Cardiopulmonary exercise testing provides valuable information about objective evidence of exercise capacity, as well as ventilatory performance and chronotropic and blood pressure responses during exercise; however, it may not be commonly performed in clinical practice. The current data suggest that clinicians might use the H_2_FPEF score to identify HFpEF with impaired exercise tolerance among patients with exertional dyspnea. This might also help determine patients who need aggressive treatment. Exercise capacity (VO_2_) is determined by cardiac (CO) and peripheral (A-VO_2_ diff) components of O_2_ transport. We demonstrated that the H_2_FPEF and HFA-PEFF scores did not correlate with A-VO_2_ diff during peak exercise. However, we cannot exclude the possibility that the HFpEF scores predict worsening physical condition, and further study is required to elucidate the supposition using other indices, such as clinical facility score.

The association between the H_2_FPEF score and peak VO_2_ may raise the question of whether therapies targeting the components of the score could improve aerobic capacity in patients with HFpEF. Obesity may be a promising target given its high prevalence and pathophysiologic significance^14^. Kitzman and colleagues demonstrated that weight loss induced by caloric restriction or aerobic exercise training improved peak VO_2_, reduced LV mass and inflammatory markers, and enhanced the quality of life in obese patients with HFpEF^23^. Bariatric surgery has been demonstrated to improve functional capacity in obese patients with HF and a reduced EF^24^. Obesity and increased adiposity may better respond to sodium-glucose co-transporter 2 inhibitors by reducing the plasma volume and visceral and epicardial fat. A substantial proportion of patients with HFpEF develop AF, and they may experience biatrial dysfunction, a poor functional capacity, RV dysfunction, and an increased risk of death^12,25,26^. Catheter ablation may be effective to reverse or at least prevent the adverse consequences of AF^27,28^ however, this should be tested in prospective trials. Intensive treatment of isolated hypertension was shown to be effective for the prevention of the development of HF^29^. Further studies are required to test whether it will prevent or mitigate the progression of HFpEF.

The current study has several limitations. All participants were referred for exercise stress echocardiography. This might introduce selection bias. The sample size was relatively small, which could cause bias in the overall results. The control group was not standard as they were referred for exercise stress echocardiography for the evaluation of exertional dyspnea and had multiple comorbidities, which could also cause bias. Given the presence of exertional dyspnea and comorbidity burden, control subjects may be considered as pre-HFpEF, and the inclusion of controls in the overall analyses might add great insight into the continuous relationships between the magnitude of HFpEF algorithms, exercise intolerance, and cardiac abnormalities across the spectrum from risk to frank HFpEF. Three different definitions were used for elevations in LV filling pressure in the HFpEF diagnostic criteria (invasively measured pulmonary capillary wedge pressure [PCWP] at rest and/or with exercise, the ASE/EACVI criteria, and exercise E/e′ ratio). There might be heterogeneity across the different definitions, which could cause bias in the results^30^. Most of the participants fulfilled Step 1 of the HFA-PEFF score (Initial workup); however, the last two steps proposed by the HFA-PEFF score (Step 3 [Advanced workup using exercise testing] and Step 4 [Etiological workup]) were not considered in this study. LV longitudinal strain was determined using apical four-chamber views. We did not have data on anaerobic threshold, which precluded assessment of the relationship between the HFpEF scores and worsening physical condition.

### Conclusions

Both H_2_FPEF and HFA-PEFF scores were associated with a lower LV systolic and diastolic function, higher estimated LV filling pressure, lower CO, and reduced RV systolic function during peak ergometry exercise. Despite the requirement of only a few input variables, the H_2_FPEF score provided a good discriminatory ability for identifying poor exercise capacity among patients with dyspnea; however, the HFA-PEFF score did not. These data may provide new insights into the role of the H_2_FPEF and HFA-PEFF scores for predicting exercise intolerance and abnormal hemodynamics in patients presenting with exertional dyspnea.

## Methods

### Study population

This was a retrospective cross-sectional study to determine the accuracy of the HFpEF scores for predicting exercise capacity and echocardiographic findings of hemodynamics during exercise. Consecutive patients who were referred to the echocardiographic laboratory of the Gunma University Hospital for exercise stress echocardiography due to exertional dyspnea between September 2019 and July 2021 were enrolled. HFpEF was defined by typical clinical symptoms (dyspnea and fatigue), normal LV EF (> 50%), and objective evidence of elevated left heart filling pressures at rest and/or with exercise (at least one of the following: the ASE/EACVI-recommended echocardiographic diastolic dysfunction; E/e′ during exercise > 15; or invasively-measured PCWP at rest > 15 mmHg and/or with supine ergometry exercise ≥ 25 mmHg)^5,31,32^.

Control subjects who were also referred for exercise echocardiography because of clinical indication of exertional dyspnea were also included (controls). The control subjects were required to have no evidence of HF (criteria above). Patients with EF < 50%, significant left-sided valvular heart disease (> moderate regurgitation, > mild stenosis), infiltrative, restrictive, or hypertrophic cardiomyopathy, and non-Group II pulmonary artery hypertension or exercise-induced pulmonary hypertension without elevation in the E/e′ ratio were excluded. The study was approved by our Institutional Review Board with the waiver of consent because of its retrospective design (Gunma University Hospital, Clinical Research Review Board), and was performed in accordance with the Declaration of Helsinki. All authors have read and agreed to the manuscript as written.

### Assessment of cardiac structure and function

Two-dimensional and Doppler echocardiography was performed by experienced sonographers using a commercially available ultrasound system (Vivid E95, GE Healthcare, Horten, Norway). EF and mitral s′ velocity were measured to assess LV systolic function. LV deformation analyses were also performed offline with the commercially available software (EchoPAC, GE, Milwaukee, Wisconsin) to measure LV longitudinal strain. An apical four-chamber view was used to calculate the LV longitudinal strain. E, e′, and the average E/e′ ratio were used to assess LV diastolic function^31^. Stroke volume was determined from the LV outflow dimension and the pulse Doppler profile, and CO was then calculated from the product of heart rate and stroke volume. PASP was calculated as 4 × (peak tricuspid regurgitation [TR] velocity)^2^ + estimated RAP. RV systolic function was assessed using systolic tissue velocities at the lateral tricuspid annulus.

All subjects underwent supine cycle ergometry echocardiography, starting at 20 W for 5 min, increasing 20 W increments in 3-min stages to subject-reported exhaustion as previously described^33^. Echocardiographic images were obtained at baseline and during all the stages of exercise. During exercise, mitral annular tissue velocities were measured at the septal annulus. All the Doppler measurements represent the mean of ≥ 3 beats. All studies were interpreted offline and in a blinded fashion by a single investigator (M.O.). In a subset of participants, an expired gas analysis was performed simultaneously with echocardiography at rest and throughout the exercise to measure breath-by-breath VO_2_. In the current study, impaired exercise capacity was defined by peak VO_2_ of < 14 mL/min/kg based on a previous study^34^. The A-VO_2_ diff was calculated by using the Fick equation (VO_2_ divided by CO)^2^.

### Calculation of the H_2_FPEF and HFA-PEFF scores

The H_2_FPEF score is based on four clinical parameters (BMI > 30 kg/m^2^ [2 points], treatment with two or more antihypertensive medicines [1 point], AF [3 points], and age > 60 years [1 point]) and two echocardiographic variables (E/e′ ratio > 9 [1 point] and PASP > 35 mmHg [1 point])^5^. This results in a categorical H_2_FPEF score ranging from 0 to 9^5^. The H_2_FPEF scores of 0–1 are associated with a low probability of HFpEF (< 25%) and the score of 6–9 is associated with a high probability of HFpEF (> 90%).

The assessment of the HFA-PEFF score was limited to Step 2 of the algorithm^6^. The score was calculated as the sum of echocardiographic functional (age-specific cut-offs for e′ velocity, E/e′ ratio, TR velocity, and longitudinal strain: maximum 2 points) and the morphological domains (rhythm-specific LA volume, relative wall thickness, and sex-specific measures of LV mass: maximum 2 points) and natriuretic peptide domains (maximum 2 points). Patients with a total score of 0 to 1 are considered to have a low probability of HFpEF, 2 to 4 as intermediate, and 5 to 6 as high probability. The HFA-PEFF score was calculated if all three domains were available.

### Statistical analysis

Data are reported as mean (SD), median (IQR), or number (%) unless otherwise specified. Between-group differences were compared using the unpaired t-test, Wilcoxon rank-sum test, or chi-square test, as appropriate. Pearson’s (normally distributed data) or Spearman’s correlation coefficients (non-normally distributed data) were used to assess relationships between two variables of interest, as appropriate. Multiple linear regression models were then used to adjust for the age, sex, BMI, and presence of hypertension. Meng’s test was used to determine whether there was a significant difference in the strength of the correlation coefficient. Receiver operating curves were constructed to evaluate the performance of HFpEF diagnostic schemes for predicting reduce exercise capacity (peak VO_2_ < 14 mL/min/kg)^34^. All tests were 2-sided, with a value of *p* < 0.05 considered significant. All statistical analyses were performed with JMP 14.0.0 (SAS Institute, Cary, NC, USA).

## Supplementary Information

Supplementary Information.
