# Supplementary material for: The H2FPEF and HFA-PEFF algorithms for predicting exercise intolerance and abnormal hemodynamics in heart failure with preserved ejection fraction
Source: Sci Rep. 2022 Jan 7;12:13. doi: 10.1038/s41598-021-03974-6 (PMC8742061; doi:10.1038/s41598-021-03974-6)
Supplement: Supplementary file 1 — Supplementary Information. [file 41598_2021_3974_MOESM1_ESM.docx]

**Supplemental Table 1. Comparisons of the Clinical Profiles, the HFpEF Scores, and the Exercise Capacity across the Different Definitions of HFpEF**

| **HFpEF (n=83)** | **Catheterization**  **(n=26)** | **ASE/EACVI**  **(n=24)** | **Ex E/e’**  **(n=33)** | **P value** |
| --- | --- | --- | --- | --- |
| Age (years) | 74±9 | 75±10 | 74±6 | 0.80 |
| Female, n (%) | 18 (69%) | 17 (71%) | 15 (45%) | 0.08 |
| H_2_FPEF score | 3.3±1.9 | 4.4±1.7* | 3.1±1.4^#^ | 0.01 |
| HFA-PEFF score | 4.6±1.4 | 5.3±0.8 | 4.5±1.3^#^ | 0.03 |
| Peak VO_2_ (mL/min/kg) | 12.1±3.4 | 10.6±3.4 | 11.8±3.3 | 0.44 |

*p<0.05 vs catheterization; ^#^p<0.05 vs ASE/EACVI. ASE/EACVI, the American Society of Echocardiography/European Association of Cardiovascular Imaging; Catheterization, pulmonary capillary wedge pressure at rest >15 mmHg and/or with supine ergometry exercise ≥25mmHg; Ex E/e’, ratio of the early diastolic mitral inflow velocity to early diastolic mitral annular tissue velocity during exercise >15; HFpEF, heart failure with preserved ejection fraction; and VO_2_, oxygen consumption.

**Supplemental Table 2. Distribution of the Patients according to the HFpEF Scores.**

|  | **Low** | **intermediate** | **High** | **Total** |
| --- | --- | --- | --- | --- |
| **H_2_FPEF score, points** | **0-1** | **2-5** | **6-9** |  |
| **HFpEF** | 3 | 66 | 14 | 83 |
| **Controls** | 48 | 56 | 0 | 104 |

|  | **Low** | **intermediate** | **High** | **Total** |
| --- | --- | --- | --- | --- |
| **HFA-PEFF, points** | **0-1** | **2-4** | **5-6** |  |
| **HFpEF** | 0 | 27 | 52 | 79 |
| **Controls** | 9 | 65 | 11 | 85 |

Values are number. Both high H_2_FPEF (6-9 points) and HFA-PEFF (5-6 points) scores showed very high positive predictive values (100% and 83%) to diagnose HFpEF while low scores (H_2_FPEF: 0-1 points and HFA-PEFF: 0-1 points) displayed very high negative predictive values (94% and 100%).

**Supplemental Table 3.** **Correlations of the HFpEF Scores with Echocardiographic Measures of Hemodynamics and Exercise Capacity in Patients with HFpEF and Controls.**

|  | **Controls (n=104)** | | | | **HFpEF (n=83)** | | | |
| --- | --- | --- | --- | --- | --- | --- | --- | --- |
|  | **H_2_FPEF score** | | **HFA-PEFF score** | | **H_2_FPEF score** | | **HFA-PEFF score** | |
|  | **r coefficient** | **P value** | **r coefficient** | **P value** | **r coefficient** | **P value** | **r coefficient** | **P value** |
| ***Echocardiographic measures during peak exercise*** | | | | |  |  |  |  |
| Mitral e’ (cm/sec) | -0.36 | 0.0002 | -0.39 | 0.0002 | 0.13* | 0.23 | -0.19 | 0.09 |
| Mitral s’ (cm/sec) | -0.33 | 0.0006 | -0.26 | 0.02 | -0.41 | 0.0001 | -0.39 | 0.0005 |
| E/e’ ratio | 0.39 | <0.0001 | 0.27 | 0.03 | 0.10* | 0.39 | 0.22 | 0.06 |
| Cardiac output (L/min) | -0.15 | 0.13 | -0.09 | 0.42 | -0.21 | 0.06 | -0.20 | 0.08 |
| A-VO_2_ difff (mL/dL) | 0.19 | 0.18 | -0.003 | 0.99 | -0.11 | 0.43 | -0.20 | 0.18 |
| TV s’ (cm/sec) | -0.26 | 0.01 | 0.05 | 0.67 | -0.49* | <0.0001 | -0.19 | 0.10 |
| PASP (mmHg) | 0.02 | 0.88 | 0.07 | 0.51 | 0.14 | 0.22 | 0.03 | 0.78 |
| Peak VO_2_ (mL/min/kg) | -0.33 | 0.02 | -0.05 | 0.75 | -0.39 | 0.004 | -0.30 | 0.04 |
| Exercise duration (min) | -0.27 | 0.006 | -0.26 | 0.02 | -0.25 | 0.02 | -0.12 | 0.29 |

*The correlation coefficient was significantly different from corresponding correlation in control subjects (p<0.05 by Meng’s z-test). A-VO_2_ diff, arterial-venous oxygen content difference; mitral e’ and s’, early diastolic and systolic mitral annular tissue velocity; PASP, pulmonary artery systolic pressure; TV s’, systolic tissue velocities at the lateral tricuspid annulus; and other abbreviations as in Supplemental Table 1.
